# Variability of Psychosocial Services Within the ImproveCareNow Learning Health System: Opportunities for Optimization

**DOI:** 10.1097/PG9.0000000000000349

**Published:** 2023-08-16

**Authors:** Joel B. Winnick, Noel Jacobs, Jennie G. David, Mai Ku Moua, Shehzad A. Saeed

**Affiliations:** From the * Geisinger Commonwealth School of Medicine; † Department of Psychiatry and Behavioral Health, Geisinger, Danville, PA; ‡ Oklahoma City University, Oklahoma, OK; § Division of Pediatric Psychology and Neuropsychology, Nationwide Children’s Hospital, Columbus, OH; ¶ Atrium Health Levine Children’s Specialty Center, Charlotte, NC; ‖ Department of Medical Affairs, Dayton Children’s Hospital, Dayton, OH; ** Boonshoft School of Medicine, Wright State University

**Keywords:** support, pediatric, inflammatory bowel disease

## Abstract

Care for youth with pediatric inflammatory bowel disease (IBD) is the focus of ImproveCareNow (ICN), an international learning health system devoted to quality care and improved outcomes through collaboration, data sharing, and research. Known to be significantly disruptive to normative social development and quality of life, pediatric IBD significantly increases the risk of internalizing distress and secondary developmental sequelae. While multidisciplinary support including psychosocial care (from social workers and pediatric psychologists) is growing, this evidence-based and beneficial set of services is not universally available to youth with IBD. In a survey sent to the more than 100 established ICN centers, psychosocial providers attempted to identify the coverage and practice scope of psychosocial providers within the network. Results indicated that support varies widely by service type and availability of providers. Recommendations for further research and considerations for centers seeking to expand supports are considered.

**What Is Known**

- ImproveCareNow (ICN) recommends Inflammatory Bowel Disease (IBD) Centers of Excellence apply a multidisciplinary team approach including specialized psychosocial services, yet most IBD care centers are lacking adequate psychosocial services.

**What Is New**

- The majority of ICN centers responding reported the presence of psychosocial services; however, nearly half reported availability of these services was limited to one day per week or less.
- Only 10 out of 51 ICN centers responding reported offering specific psychosocial services devoted exclusively to IBD care.
- The amount of time pediatric patients with IBD and their families may have access to psychosocial services remains limited even for centers with psychosocial service availability.

Youth with inflammatory bowel disease (IBD) are known to be at elevated risk to experience depression, anxiety, social difficulties, poorer school functioning, lower health-related quality of life, fatigue, body image complexities, and medical trauma (1–3). Psychological distress in pediatric patients with IBD has also been associated with nonadherence (4), which may negatively impact medical outcomes and subsequent increased utilization of medical services (5). Patient and family psychosocial factors including difficulties coping with symptoms of anxiety and depression are associated with increased report of pain and disability among youth with IBD (6). The COVID-19 pandemic has also impacted psychosocial needs in pediatric patients with IBD, with pandemic-related disruptions negatively contributing to both caregiver and child well-being (7). Taken together, there has been a call for increased engagement with psychosocial providers and routine mental health screenings for patients throughout pediatric IBD care given the dynamic nature of psychosocial needs of patients and caregivers alike (1–13).

The ImproveCareNow (ICN) Network is an international learning health system with the goal of improving the health of youth with IBD (14). ICN promotes best practices in caring for youth with IBD by engaging with key invested partners including patients and families, medical providers, nurses, psychosocial providers, researchers, dieticians, nutritionists, and technical and communications professionals. A standing committee of this learning health system, the ICN Community Council, includes representative members of all the key invested partner groups of the community and is co-chaired by a physician and a parent. ICN has published recommendations for IBD Centers of Excellence identifying specialized psychosocial services as an essential component of a multidisciplinary team, yet most IBD care centers are lacking adequate psychosocial services for pediatric patients with IBD (15). Psychosocial health needs have repeatedly been emphasized as important and needed for quality IBD care by patient and family advocates within ICN, which continues to highlight the patient- and family-driven interest in having whole-person care in this disease population (16).

The aim of this work was to better understand the availability of psychosocial services across ICN centers to directly inform effective advocacy efforts for programmatic development for these services and identify opportunities to optimize these psychosocial services across the pediatric IBD care continuum. We consider this to be a baseline assessment of available psychosocial resources, and if available, variations in kinds of services (eg, assessment, intervention), staffing roles and easy accessibility of these services in both an ambulatory and inpatient setting.

## METHODS

### Procedures

Following a request from the ICN Community Council to better understand availability of psychosocial supports across the network, several members of the Social Work and Psychology (SWAP) workgroup in collaboration with the ICN Communications team conducted a brief survey through the secure Jotform platform of our ICN Care Center Points of Contact (POC; Center Coordinators were typically not physicians) for each of the 102 ICN care centers including 96 US and 6 international centers at the time of the survey.

### Data Collection

**FIGURE 1.**
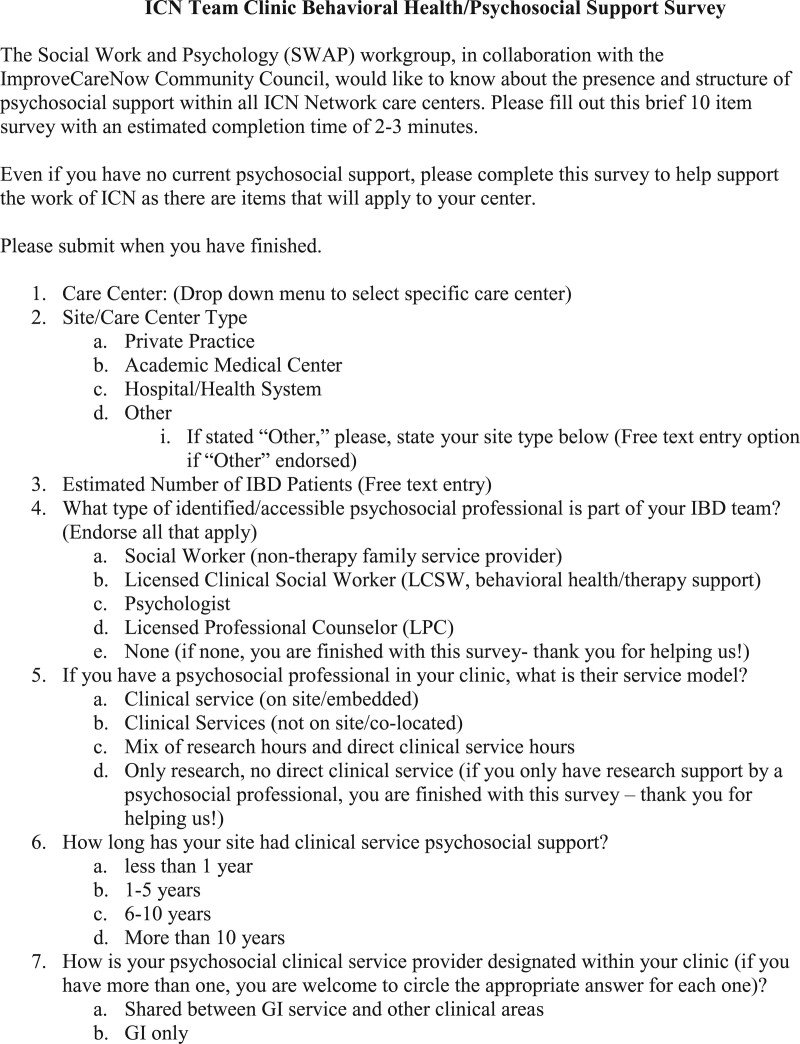
ICN team clinic behavioral health/psychosocial support survey. ICN = ImproveCareNow.

The 10-item survey appears in Figure 1. No private health information was collected through this survey. The survey opened May 10, 2022, through a message via the ICN Communications team to the ICN Digest listserv which is a twice monthly newsletter sent out to the whole ICN community. Due to a low initial response rate of 24.5% (25/102 centers), the survey was extended until June 17, 2022. The ICN Communications team then sent targeted messages to all ICN Care Center POCs identified as site coordinators. To maximize the response rate, SWAP members on the ICN information hub were directly encouraged to contact POCs at their Centers to ensure inclusion. Through this intervention, there was a survey response rate increase from 24.5% (25/102) to 56.9% (58/102). For multiple entries per center or instances of missing data, POCs were contacted for data cleaning and clarification. The survey closed on July 5, 2022.

Survey data were exported into Microsoft Excel for analysis. While the goal was for all centers to respond, the final survey response rate of 56.9% was deemed adequate as the National Institutes of Health *Quality Assessment Tool for Observational Cohort and Cross-Sectional Studies* specifies participation rates of ≥50% are needed to adequately represent the target population (17). In total, 58 US centers responded to this current survey out of 96 US and 6 international centers. Following the data of cleaning procedures described above, there was a total of only 6 remaining center responses with any missing data points (eg, 3 did not include patient population estimates, 1 did not include center name, 1 did not identify center type, and 1 did not answer items concerning access or clinical services provided) representing an overall percentage of 89.7% of complete responses.

## RESULTS

### Descriptive Statistics

Of the data available representing 58 US centers, 52 (89.7%) had some form of psychosocial services. Responding centers included academic medical centers (n = 31, 53.4%), health systems (n = 23, 39.7%), private practices (n = 3, 5.2%), and unidentified (n = 1, 1.7%). Based upon survey responses (n = 55), the average estimated number of patients receiving care per Center was 372.2 (SD = 287.3, Mdn = 275.0, Mode = 200.0, range = 100–1600). Please see Table 1 for data regarding the presence of services across centers.

**TABLE 1.** Availability of psychosocial services across ImproveCareNow network care centers

Survey responses (n)	Percent endorsed (n)
Psychosocial professional is part of your IBD team (58)^*^	
Licensed clinical social worker (behavioral health/therapy support)	39.7% (23)
Social Worker (nontherapy family service provider)	50.0% (29)
Psychologist	63.8% (37)
Licensed professional counselor	1.72% (1)
None	10.3% (6)
Psychosocial professional service model (47)	
Clinical service (onsite/embedded)	93.6% (44)
Clinical services (not on site/co-located)	2.1% (1)
Mix of research hours and direct clinical service hours	4.3% (2)
Only research, no direct clinical service	0.0% (0)
Duration of psychosocial support on site in years (50)	
<1	0.0% (0)
1–5	54.0% (27)
6–10	18.0% (9)
>10	28.0 % (14)
Psychosocial professional designation within your clinic (51)^*^	
Shared between GI service and other clinical areas	47.1% (24)
Gastrointestinal only	43.1% (22)
IbD only	19.6% (10)
Access to psychosocial professional for patients (48)	
0%–10% (half day)	25.0% (12)
11%–20% (up to one full day or two half-days)	22.9% (11)
21%–50%	20.8% (10)
51%–80%	6.3% (3)
81%–95%	8.3% (4)
96%–100%	16.7% (8)
Clinical services provided (50)^*^	
Brief consult only	42.0% (21)
Brief targeted care with limited number of sessions	62.0% (31)
Multidisciplinary clinic same day care with all IBD clinic specialties	62.0% (31)
Behavioral health evaluation and routine return visits	56.0% (28)
Outpatient and inpatient continuity of care over time	46.0% (23)
Psychosocial services via telehealth (48)	
0%–10%	39.6% (19)
11%–25%	18.8% (9)
26%–50%	14.6% (7)
51%–75%	20.8% (10)
76%–99%	2.1% (1)
100%	4.2% (2)

IBD = inflammatory bowel disease.

^*^Respondents were directed to endorse all that apply. Total percentage values for these items will be >100% as the denominator used to calculate the percentage was the number of centers responding to each respective item.

## DISCUSSION

Recommendations for IBD Centers of Excellence include a multidisciplinary team with specialized psychosocial services (15), which also aligns with calls for an increase in engagement with psychosocial services for pediatric patients with IBD and their families (1–13,16). Data derived from this survey denoted that out of 58 US centers responding, 52 (89.7%) have identifiable available psychosocial services for pediatric patients with IBD. Most of the responding centers had psychosocial services; however, the availability of those services for clinical application were limited to 1 day/week or less for nearly half of the centers responding (47.9%), with the majority of responding centers (68.8%) reporting psychosocial services being available only half or less of the time per week. This is notable as the average count of patients per center was 372 with median and mode values of 275 and 200, respectively. Given the number of patients treated across centers, the amount of time patients may have access to psychosocial services likely remains inadequate.

Of those centers with available data, psychosocial service providers were predominately social workers, followed by psychologists, and a single licensed professional counselor. Most centers with psychosocial services had 2 psychosocial providers, yet more than one-third had only one. Most psychosocial services were embedded clinical services with only 2 instances of providers with a mix of research and clinical hours and one instance of offsite clinical services.

For those centers with identified psychosocial services, only 10 of 51 had exclusive IBD only psychosocial services. Most centers with psychosocial services for youth with IBD had those services shared within the broader gastrointestinal department or across multiple clinical areas, such that the psychosocial providers did not focus primarily of IBD. More than half of centers reported having multidisciplinary care clinics or brief targeted care with approximately one third offering both, yet less than half of responding centers reported having outpatient and inpatient continuity of psychosocial services.

Additionally, most centers with psychosocial supports reported only having this service for 5 years or less, suggesting psychosocial services were generally new for most centers. Most psychosocial service providers also incorporated some degree of telehealth, with more than one quarter offering this service for more than half of all visits. While telehealth may be useful for patients and families in accessing this service, especially during the COVID-19 pandemic, more than one-third of psychosocial services utilized telehealth 10% or less which might also suggest that many psychosocial providers have either resumed in person services or are in the process of determining how to optimize this modality of clinical care.

There were notable limitations for this study. Although the survey response rate was favorable, it does not represent the entire ICN network and may represent a lower bound estimation of psychosocial services available across ICN centers. We were unable to determine the availability of psychosocial services at the 44 centers that did not respond to the survey. Nonetheless, through efforts to directly contact POCs for ICN care centers and SWAP workgroup members, the survey response rate increased from 24.5% (25/102) to 56.9% (58/102). Even with a response rate of ≥50% being considered as adequate to represent the target population (17), an increased response rate for future surveys would be desirable. The data present are also limited to only US centers. While not assessed through this survey, the paucity of psychosocial services furthers concern for equity in access across racial, ethic, and insurance considerations for families. As such, efforts to gather data regarding equity, diversity, inclusion should be considered in future surveys. Surveys were completed primarily by center coordinators serving as POCs for their clinic and provided estimates, nevertheless in most cases psychosocial providers were not contacted directly unless there was a non-response from their clinic or responses needed to be clarified through the data cleaning process. Since respondents were asked to identify Center type, this approach did not allow for an analysis of differences in survey response rates based upon the center type as data were not available for nonresponding centers, which was an additional limitation. The survey did not directly assess the availability of other resources that may support psychosocial care in pediatric IBD, such as a support group. For the sake of brevity, this study did not assess wait time. Future research should consider assessing wait time to gain further understanding of access to psychosocial care. Notwithstanding being outside of the scope of this current study, future studies might examine value-added factors such as improving patient outcomes or cost-savings through multidisciplinary IBD team collaborations with psychosocial services as evidenced by reduced emergency department utilization, better adherence to treatment recommendations, and improved shared decision-making.

Opportunities to optimize psychosocial services across the pediatric IBD care continuum will likely need to include efforts to increase integration of psychosocial care through ongoing discussions related to funding, support leadership at institutions in appreciating the importance and role of psychosocial care in this population, address stigma related to mental health utilization, educate patients and caregivers on potential benefits of psychosocial services, increase exposure to psychosocial care in IBD, and a possible movement toward an “opt-out” versus an “opt-in” model in recognizing the normative psychosocial needs of IBD patients and families (11). Recommendations from a recent paper discussing integration of psychosocial services in pediatric specialty care also included identifying a physician champion to support integration efforts and pediatric psychologists increasing their profiles and engagement with medical providers (18).

The aim of this study was to effectively utilize survey data to examine the gap between the general calls or the perceptions of a need for more psychosocial services in pediatric IBD care (15) and quantify the limited availability of psychosocial services across ICN care centers to inform advocacy efforts. Indeed, while the majority of responding ICN care centers had a psychosocial service presence, the current availability of those services remains limited. Psychosocial distress can impact quality of life, disability status, medical outcomes, and utilization of medical services for youth with IBD, and there is a palpable need for psychosocial services, routine patient mental health screenings, and related supports for this at-risk population (1–13). Efforts to bolster advocacy for psychosocial services will likely need to occur through continued collaborations between invested partners including medical providers, system level leaders, researchers, psychosocial providers, and patients and their families.

## ACKNOWLEDGMENTS

The authors greatly appreciate the work of the ICN Communications Team including Julie Massie, Melissa Mock, Mary Havens, and Jen Savas for their support of this project.
